# Hormonal and metabolic adaptations in Omega-3-supplemented rabbit does during their first reproductive cycle

**DOI:** 10.1080/01652176.2026.2749322

**Published:** 2026-10-29

**Authors:** Alda Quattrone, Nour Elhouda Fehri, Olimpia Barbato, Giovanni Ricci, Daniele Vigo, Luigj Turmalaj, Majlind Sulçe, Marta Castrica, Maria Laura Marongiu, Alessandro Dal Bosco, Bayrem Jemmali, Gerald Muça, Gabriele Brecchia, Giulio Curone, Laura Menchetti

**Affiliations:** a Department of Veterinary Medicine and Animal Sciences, University of Milan, Lodi, Italy; b Department of Veterinary Medicine, University of Perugia, Perugia, Italy; c Faculty of Veterinary Medicine, Agricultural University of Tirana, Tirana, Albania; d Department of Life Science, Health and Health Professions, Università degli Studi “Link Campus University”, Rome, Italy; e Department of Veterinary Medicine, University of Sassari, Sassari, Italy; f Department of Agricultural, Food and Environmental Science, University of Perugia, Perugia, Italy; g LR13AGR02, Mateur Higher School of Agriculture, University of Carthage, Mateur, Tunisia; h School of Biosciences and Veterinary Medicine, University of Camerino, Matelica, Italy

**Keywords:** Extruded linseed, *Padina pavonica*, insulin, leptin, cortisol, thyroid hormones, NEFA, glucose

## Abstract

This study evaluated the effects of dietary *n*-3 polyunsaturated fatty acids (*n*-3 PUFA) supplementation on endocrine and metabolic profiles of forty-five nulliparous rabbit does during their first reproductive cycle. Animals were randomly assigned to one of three diets: control (CNT), 5% extruded linseed (L), and 5% extruded linseed plus 0.2% *Padina pavonica* extract (LPP). Blood was sampled at baseline, artificial insemination, parturition, mid-lactation, and weaning, to determine the concentrations of cortisol, insulin, leptin, thyroid hormones (T_3_, T_4_), glucose, and non-esterified fatty acids (NEFA). Reproductive stage significantly influenced endocrine and metabolic dynamics, with cortisol and NEFA peaking at parturition (*p* < 0.001). Supplemented groups, particularly LPP, showed higher T_3_ concentrations (*p* < 0.05) and T_3_/T_4_ ratios (*p* < 0.01). The L diet maintained higher insulin during lactation (*p* < 0.05), while LPP lowered leptin at parturition (*p* < 0.01) and NEFA at insemination (*p* < 0.01), indicating an early decrease in lipid mobilization. Multivariate analysis suggested that *n*-3 PUFA supplementation promoted an endocrine profile characterized by enhanced thyroid activity and reduced lipid mobilization, consistent with a potentially favorable metabolic shift. In conclusion, enriching diets with *n*-3 PUFA, especially linseed combined with *Padina pavonica* extract, represents a promising nutritional strategy to support rabbit does during the highly demanding phases of their first reproductive cycle.

## 1. Introduction

The welfare and productivity of breeding rabbit does are intrinsically linked to their capacity to cope with the substantial metabolic demands imposed by the reproductive cycle (Munari et al. 2020). Both pregnancy and lactation require profound metabolic and endocrine adaptations to maintain maternal energy homeostasis while simultaneously supporting fetal growth, mammary development, and milk synthesis (Menchetti et al. 2020). In prolific species such as rabbits, these requirements are particularly intense due to large litter sizes and the high energetic cost associated with rapid neonatal development (Arias-Álvarez et al. 2009). These demands are particularly relevant in nulliparous does, which enter their first reproductive cycle while still undergoing somatic growth (Martínez-Paredes et al. 2012). Typically bred upon reaching about 75% of their mature body weight, these young females must partition available nutrients among their own continued physical development, fetal growth, and the maturation of reproductive tissues, including the uterus and mammary glands (Martínez-Paredes et al. 2018). Confronted with this extreme metabolic pressure, the accelerated mobilization of body reserves can trigger significant losses in body weight and muscle mass, elevate oxidative stress, and cause systemic metabolic dysregulation (Lozano et al. 2019; Peiró et al. 2023). Ultimately, these alterations can impair ovarian function, oocyte maturation, embryo survival, and milk production (Castellini et al. 2010; Saidj et al. 2018). Consequently, failing to adapt to these dynamic metabolic demands severely compromises fertility and shortens the productive lifespan of the doe, representing a major threat to both reproductive efficiency and animal welfare (Rosell et al. 2020; Pinto-Pinho et al. 2023).

For these reasons, evaluating hormonal and metabolic parameters across critical reproductive phases provides essential insights into the adaptive resilience and welfare of breeding does (Menchetti et al. 2020). These endocrine biomarkers serve as central regulators of energy homeostasis, capturing the complex interplay among dietary intake, systemic metabolism, and reproductive activity (Garcia-Garcia 2012). Notably, their circulating concentrations fluctuate throughout the reproductive cycle to accommodate the changing physiological demands of mating, gestation, and lactation (Menchetti et al. 2015).

Specifically, cortisol serves as a primary indicator of physiological stress and a key modulator of energy homeostasis, promoting gluconeogenesis, lipolysis, and protein catabolism to ensure substrate availability during periods of increased metabolic demand (Eckersall and Bell 2010; Accorsi et al. 2017). Leptin, a peptide hormone predominantly produced by adipose tissue, acts as a major metabolic signal of energy sufficiency, exerting anorexigenic and catabolic effects while linking nutritional status to reproductive function through modulation of the hypothalamic–pituitary axis and direct actions on the gonads (Chan and Mantzoros 2001; Tena-Sempere 2007; Sirotkin et al. 2009). As the principal anabolic hormone, insulin coordinates nutrient partitioning and systemic glycemic control by promoting cellular glucose uptake (primarily in skeletal muscle and adipose tissue), stimulating glycogenesis, lipogenesis, and protein synthesis, while simultaneously inhibiting hepatic gluconeogenesis and lipolysis (Chadt and Al-Hasani 2020; Ladyman and Brooks 2021). Furthermore, thyroid hormones (triiodothyronine, T_3_; and thyroxine, T_4_) regulate the basal metabolic rate, supporting the physiological adaptations required for gestation and fetal development (Forhead and Fowden 2014; Rodríguez-Castelán et al. 2017). Among the circulating metabolites, blood glucose reflects immediate energy availability, whereas non-esterified fatty acids (NEFAs) are markers of lipid mobilization and negative energy balance (Calle et al. 2017; Veenstra et al. 2020). Evaluated collectively, these endocrine and metabolic biomarkers can provide an integrated characterization of the metabolic profile of breeding does, offering a more comprehensive assessment of physiological adaptation than reproductive performance indicators alone.

Appropriate nutritional strategies are essential for preserving metabolic balance throughout the demanding phases of the reproductive cycle (Goswami, 2025). Evidence from both human and animal studies demonstrates that dietary imbalances during pregnancy, whether involving undernutrition or nutrient excess, can negatively impact maternal physiology and subsequent offspring development, ultimately impairing fetal viability, growth rates, and long-term metabolic programming of newborns (Innis 2011; Hadjadj et al. 2025). Therefore, modern dietary strategies should not only meet quantitative nutrient requirements but also support metabolic resilience and endocrine stability. In this context, there is growing interest in functional feed ingredients capable of modulating metabolic and inflammatory pathways to enhance welfare and productivity in rabbit production systems (El-Sabrout et al. 2023; Abdelsalam and Fathi 2023). Omega-3 polyunsaturated fatty acids (*n*-3 PUFAs) are particularly noteworthy for their well-established anti-inflammatory, antioxidant, and metabolic effects (Simopoulos 2002; Calder 2010). In rabbit does, dietary supplementation with extruded linseed, a rich source of *α*-linolenic acid (ALA, 18:3 *n*-3), has been shown to improve fertility, sexual receptivity, and steroidogenesis, while also enhancing litter size and viability (Habeeb et al. 2021; Agradi et al. 2023; Quattrone et al. 2024). Beyond their multiple roles in reproduction, *n*-3 PUFAs can influence hormonal and metabolic pathways by improving insulin sensitivity (Sinha et al. 2023; Albardan et al. 2024) and modulating leptin signaling (Jazinaki et al. 2024; Jerab et al. 2025).

More recently, marine macroalgae have emerged as sustainable and multifunctional feed ingredients within the livestock sector (Al-Soufi et al. 2022; Kazemi 2025). The brown alga *Padina pavonica*, in particular, is characterized by a complex bioactive profile that encompasses long-chain *n*-3 PUFAs, phlorotannins, polysaccharides, carotenoids, vitamins, and essential minerals (Can and Sanlier 2026). These compounds exhibit strong antioxidant and anti-inflammatory properties that may contribute to the maintenance of redox balance, immune function, and metabolic regulation (Makhlof et al. 2024). Thus, the inclusion of *Padina pavonica* in rabbit diets could potentially offer an innovative approach to combining environmental sustainability with targeted physiological benefits, although precise dose-response evaluations for this species remain necessary.

Despite the growing body of research on *n*-3 PUFA supplementation in rabbit nutrition, limited attention has been devoted to its longitudinal effects on the endocrine and metabolic profiles of breeding does throughout the reproductive cycle. Based on these considerations, we hypothesized that dietary supplementation with extruded linseed, either alone or in combination with *Padina pavonica* extract, would influence endocrine and metabolic responses associated with the first reproductive cycle in rabbit does. Therefore, the aim of this study was to evaluate the effects of these nutritional strategies on key endocrine and metabolic indicators throughout the first reproductive cycle, thereby providing further insight into the physiological adaptations of rabbit does during reproduction.

## 2. Materials and methods

### 2.1. Animals, diets, and experimental design

Forty-five nulliparous New Zealand White rabbit does, entering the study at four months of age, were housed at a commercial rabbit breeding facility. The animals were monitored throughout their first reproductive cycle. This management strategy was adopted to prevent the metabolic overlap between gestation and lactation, which commonly occurs under intensive rabbit production systems. Rabbits were individually housed in standard wire-mesh cages (75 × 38 × 25 cm; length × width × height) under controlled environmental conditions, with ambient temperature between 18 and 23 °C, relative humidity of 60-75%, and a photoperiod of 16 hours of light and 8 hours of darkness.

Upon enrollment, the female rabbits were randomly assigned to three experimental groups (*n* = 15/group) and fed accordingly three different pelleted diets. Prior to the onset of the reproductive cycle, the animals underwent a 60-day nutritional adaptation period to ensure full acclimation to the experimental diets (Figure 1). The diets, previously validated and described in earlier experiments (Quattrone et al. 2024), were formulated to provide comparable crude protein concentrations and similar calculated digestible energy, while meeting the nutritional requirements for breeding rabbit does (Blas and de Wiseman, 2010). The control group (CNT) received a standard commercial basal diet, while the other two groups received modified diets in which part of the ingredients of the commercial diet were replaced with 5% extruded linseed (L) and 5% extruded linseed plus 0.2% *Padina pavonica* algae extract (LPP), respectively (Table 1, as reported in Quattrone et al. (2024)). Proximate chemical composition of the experimental diets was determined according to standard AOAC methods and is reported in Table 2 (Soest and Wine 1967). The fatty acid profile was analyzed by gas chromatography following conversion of lipid extracts to fatty acid methyl esters (FAMEs) according to established protocols (Table 3) (Quattrone et al. 2024; Fehri et al. 2025).

A restricted feeding regimen was applied to match the physiological demands of the reproductive cycle (Blas and de Wiseman, 2010). At the beginning of the experimental period, rabbits received an initial daily ration of 130 g per animal. Following artificial insemination, the feed allowance was progressively increased by approximately 30 g per week throughout gestation to meet the increasing nutritional requirements. During lactation, the feed allowance was further adjusted according to litter size, reaching a maximum average allowance of approximately 450 g/day per doe. Fresh and clean drinking water was provided ad libitum throughout the entire experimental period via automatic nipple drinkers.

**Table 1.** Ingredient composition (g/kg) of the experimental diets: CNT, control diet; L, diet supplemented with 5% extruded linseed; LPP, diet supplemented with 5% extruded linseed and 0.2% *Padina pavonica* extract.

Ingredients (g/kg)	CNT	L	LPP
Wheat bran	250.7	248.7	249.1
Barley	133.3	130	130
Sunflower seed meal	120	116.7	115
Alfalfa	108.3	130	130
Sunflower husks	100	100	100
Beet pulp	75	56.6	55
Extruded linseed	–	50	50
Full-fat soybean	50	29.5	31
Wheat straw	41.7	20	20
Sugarcane molasses	30	30	30
Wheat	25	25	25
Grape seeds meal	23.3	16.7	16.7
Soya hulls	0	1.67	1.67
Calcium carbonate	16	14.8	14.2
Soybean oil	7.8	–	–
Sodium chloride	4	4	4
Palm oil	3.3	–	–
Carboxymethylcellulose	3	3	3
Oligo vitamin supplement^1^	2.5	2.5	2.5
*Padina pavonica* algae extract	–	–	2
Lysine HCl	1.6	1.7	1.7
Liquid acidifier^2^	1.5	1.5	1.5
Magnesium oxide	1	1	1
Methionine hydroxyanalog	0.6	0.7	0.7
Liquid choline	0.5	0.5	0.5
Vitamin E 50%	0.3	0.3	0.3
L Threonine	0.3	0.1	0.1
DL Methionine	0.3	–	–

^1^ Vitamin-mineral premix composition: Vitamin A 6,000,000 IU, D3 600,000 IU, E 20,000 IU, K3 1200 mg, B1 800 mg, B2 1600 mg, B6 800 mg, B12 6.0 mg, Biotin 60.0 mg, Niacinamide 16,000 mg, Folic acid 400 mg, Calcium pantothenate 6666 mg.

^2^ Liquid acidifier composition: Formic acid 75%.

**Table 2.** Proximate chemical composition (g/kg) of the experimental diets: CNT, control diet; L, diet supplemented with 5% extruded linseed; LPP, diet supplemented with 5% extruded linseed and 0.2% *Padina pavonica* extract.

Chemical composition (g/kg) of dry matter	CNT	L	LPP
Dry matter	893.4	894.9	899.4
Crude protein	177.2	183.3	185.9
Ether extract	39.5	62.1	52.2
Ash	79.9	76.9	82.1
NDF^1^	432.4	396.5	424
ADF^2^	279	257.2	255.3
ADL^3^	77.6	73.8	72.2
DE^4^	9.8324	9.8324	9.8324

^1^ NDF: neutral detergent fiber (%);

^2^ ADF: acid detergent fiber (%);

^3^ ADL: acid detergent lignin (%).

^4^ DE: digestible energy (MJ/kg), estimated according to Maertens et al. (1988).

**Table 3.** Fatty acid profile (g/100 g of total fatty acids) of the experimental diets: CNT, commercial control diet; L, diet containing 5% extruded linseed; LPP, diet containing 5% extruded linseed and 0.2% *Padina pavonica* algae extract.

Fatty acid (g/100 g of fatty acids)	CNT	L	LPP
C12:0	0.00	0.00	0.00
C14:0	0.18	0.20	0.20
C15:0	0.09	0.08	0.08
C16:0	13.92	11.14	11.65
C16:1 cis9	0.18	0.20	0.18
C17:0	0.12	0.11	0.10
C17:1	0.05	0.05	0.05
C18:0	3.07	3.82	3.48
C18:1	25.39	23.89	23.98
C18:2 cis *n*-6, LA^1^	47.63	32.41	33.53
C20:0	0.28	0.22	0.22
C18:3 *n*-6, *γ*-ALA^2^	0.21	0.12	0.19
C18:3 *n*-3, *α*-ALA^2^	6.53	23.25	22.63
C20:2	0.00	0.00	0.04
C20:3 *n*-3	0.00	0.00	0.02
C22:0	0.00	0.00	0.23
C22:1	0.00	0.00	0.04
C20:4 *n*-6, AA^3^	0.00	0.00	0.08
C22:2	0.00	0.00	0.01
C20:5 *n*-3, EPA^4^	0.00	0.00	0.13
C24:0	0.00	0.00	0.00
C24:1	0.00	0.00	0.03
C22:5 *n*-3, DPA^5^	0.00	0.00	0.05
C22:6 *n*-3, DHA^6^	0.00	0.00	0.06
Total	97.64	95.50	97.00
Others	2.36	4.50	3.00

^1^ LA: linoleic acid;

^2^ ALA: *α*-linolenic acid;

^3^ AA: arachidonic acid;

^4^ EPA: eicosapentaenoic acid;

^5^ DPA: docosapentaenoic acid;

^6^ DHA: docosahexaenoic acid.

Following the 60-day nutritional adaptation period, artificial insemination (AI) was performed when the female rabbits reached 6 months of age. Each female was inseminated with 0.2 mL of diluted (1:5) fresh heterospermic semen using a sterile insemination pipette. Ovulation was induced immediately prior to insemination by an intramuscular injection of 10 μg synthetic gonadotropin-releasing hormone (GnRH; Receptal®, Hoechst-Roussel Vet, Milan, Italy). Pregnancy was diagnosed by abdominal palpation 12 days post-insemination, and all forty-five rabbits were confirmed pregnant. After parturition, females were housed with their litters; litter size was very similar among groups (range: 6-7 kits; CNT: 6.78 ± 0.70; L: 7.67 ± 0.40; LPP: 7.29 ± 0.62 kits) and was therefore not standardized. Lactation was managed under a controlled nursing system, with nest box access restricted to once daily until day 18 post-partum. From day 18 onwards, the nest boxes were permanently opened, allowing kits unrestricted access to the maternal cage and feed, thereby marking the end of the controlled lactation phase. Rabbit kits were weaned at 35 days of age, when they were separated from their mothers.

### 2.2. Blood sampling and measurements of hormones and metabolites

Blood samples were collected from each rabbit at five time points during the experimental trial: at the beginning of the study, prior to the dietary adaptation (baseline; T0); at the time of artificial insemination (T1); at parturition (T2); at mid-lactation, corresponding to the end of the controlled lactation phase (18 days postpartum; T3); and at weaning of the rabbit kits (35 days post-partum; T4) (Figure 1). Samples were drawn from the marginal ear vein into EDTA-coated tubes, then immediately centrifuged at 3000 x g for 15 minutes. The resulting plasma was separated and subsequently stored at −20 °C until analysis of hormonal and metabolic parameters.

**Figure 1.**
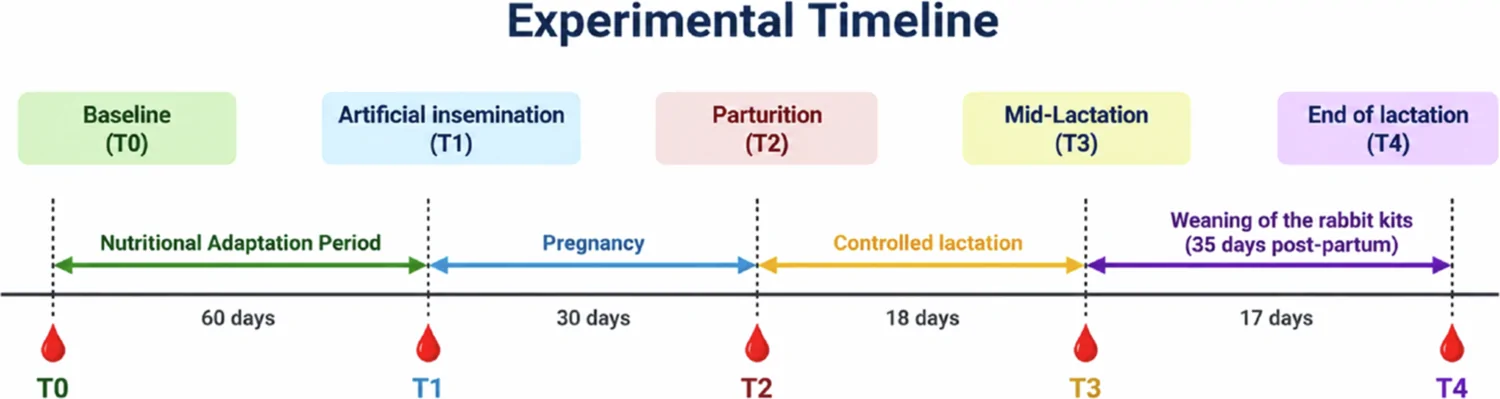
Experimental timeline and blood sampling schedule. Blood samples were collected from each rabbit at five time points: T0, baseline (prior to dietary adaptation); T1, artificial insemination; T2, parturition; T3, mid-lactation (18 days postpartum); and T4, weaning of the rabbit kits (35 days postpartum).

Cortisol, insulin, leptin, thyroid hormones (T_3_ and T_4_), non-esterified fatty acids (NEFA), and glucose were analyzed from each sample, following the methods previously described by Menchetti et al. (2020). Briefly, leptin concentrations were measured using a double-antibody RIA with a multispecies leptin kit (Linco Research Inc., St. Charles, MO, USA). Plasma insulin was quantified with a double-antibody/PEG technique with a porcine insulin RIA kit (Linco Research Inc., St. Charles, MO, USA), using guinea pig anti-porcine insulin antiserum and recombinant human insulin as the labeled antigen and standard. Total T_3_ and T_4_ were measured by RIA according to the manufacturer's instructions (Immunotech, Prague, Czech Republic), while cortisol concentrations were assessed using the CORT kit (Immunotech, Prague, Czech Republic). NEFA and glucose concentrations were analyzed following García-García et al. (2011) and Rommers et al. (2006), respectively. NEFA levels were determined using a two-reaction enzymatic-based colorimetric assay (Wako Chemicals GmbH, Neuss, Germany), and glucose concentrations were measured using the glucose oxidase method with the Glucose Infinity kit (Sigma Diagnostic Inc., St. Louis, MO, USA).

### 2.3. Statistical analysis

Diagnostic plots, including residuals versus fitted values and normal Q–Q plots, together with Kolmogorov–Smirnov tests, were used to assess model assumptions, evaluate residual distributions, identify potential outliers, and verify homoscedasticity. No substantial violations of model assumptions were detected. Insulin values were natural log (ln)-transformed for statistical analyses. Raw data are presented in the figures, whereas estimated differences and confidence intervals are reported on the transformed scale. Data were analyzed using generalized linear mixed models using a normal distribution with an identity link. Animal identity was entered as a random intercept to account for between-subject variability. Time (4 levels: T1-T4) was treated as a within-subject (repeated) factor with an unstructured covariance matrix. Group (three levels: CNT, L, LPP), time (T1-T4), and their interaction were specified as fixed effects, while baseline levels (T0) were included as covariates. Litter size was not included as a covariate because it showed limited variability (range: 6–9 kits) and did not differ significantly among dietary groups (*p* = 0.576). Therefore, its inclusion was unlikely to provide meaningful additional information while unnecessarily increasing model complexity. Post hoc pairwise comparisons were adjusted using the sequential Sidak correction. Estimated marginal means, pairwise differences, and corresponding 95% confidence intervals are reported to facilitate interpretation of biological relevance. An a priori power analysis performed using G*Power (ver. 3.1.9.4) for repeated-measures ANOVA, within–between interaction, considering three groups, four repeated measurements, and a medium effect size (f = 0.25), indicated that the total sample size of 45 animals provided high statistical power (95.5%) to detect group × time interaction effects. Nevertheless, the number of animals per group may still limit the detection of small pairwise differences, particularly after adjustment for multiple comparisons.

The correlations among hormones and metabolites were evaluated using the Pearson coefficient (r), and it was considered poor if r < │0.3│, medium if │0.3│ ≤ r < │0.5│, and large if r ≥ │0.5│.

Two discriminant analyses (DA) were performed as exploratory multivariate analyses to characterize hormonal and metabolic profiles: DA1 examined differences associated with nutritional treatment, whereas DA2 evaluated changes across physiological phases. DA was selected because, unlike univariate approaches, it derives composite variables, namely discriminant functions (DFs), that maximize separation among predefined groups and allow evaluation of whether several endocrine and metabolic variables jointly contribute to group discrimination (Pituch and Stevens 2015; Agradi et al. 2020). This multivariate approach was used to complement the univariate analyses by providing an integrated description of the hormonal and metabolic profiles associated with dietary treatment (e.g. *n*-3 PUFA supplementation) and physiological stages (such as pregnancy and lactation) that maintain homeostasis. To isolate the effect of diet, DA1 was conducted on samples collected at AI (i.e. after the nutritional adaptation period and before the onset of major physiological changes), using the experimental group as the classification variable. To investigate hormonal and metabolic dynamics across physiological states, DA2 was performed using sampling times corresponding to distinct physiological phases (i.e. AI, parturition, day 18 of lactation, and weaning) as the grouping factor. Cortisol, leptin, insulin, T_3_, NEFA, and glucose served as candidate predictors. T₄ was excluded, and T₃, the biologically active hormone, was retained to reduce predictor dimensionality and enhance model parsimony. The T₃/T₄ ratio was also excluded because it is a derived metric that is inherently collinear with T₃. Moreover, only variables showing evidence of discrimination in the preliminary Tests of Equality of Group Means (*p* < 0.05) were considered for inclusion in the final models. Homogeneity of covariance matrices was evaluated using Box’s M test, while multicollinearity among predictors was assessed by inspection of correlation matrices and tolerance/VIF values. The final set of predictors was therefore selected to balance discriminatory ability, biological relevance, and model parsimony. Discriminant functions were interpreted using structure coefficients (i.e. the correlations between the DF and the original variables); only coefficients greater than 0.4 were considered (Pituch and Stevens 2015; Zappaterra et al. 2021). Group’s centroids (i.e. mean DF score) were used to determine which functions most effectively distinguish among nutritional treatments (DA1) and physiological phases (DA2) (Pituch and Stevens 2015). The biological interpretation of DFs was made cautiously and should be considered exploratory, as discriminant functions identify patterns of multivariate separation rather than direct causal relationships among variables. Moreover, because DA does not account for the within-animal correlation arising from repeated measurements, the independence assumption was not fully met for DA2. Therefore, DA2 was used only to visualize multivariate patterns across physiological stages, whereas statistical inference regarding temporal changes was based on the mixed-model analyses, which explicitly accounted for repeated measurements within animals.

Statistical analysis was performed using SPSS 23.0 (SPSS Inc., Chicago, USA), and considered significant at a level of 0.05.

## 3. Results

### 3.1. Influence of nutritional treatment and physiological condition on hormones and metabolites

Hormonal and metabolic profiles during the different physiological stages are presented in Figures 2 and 3, respectively. The effects of basal levels, dietary treatment, physiological stage, and their interaction are summarized in Table 1SM, while detailed pairwise comparisons among dietary treatments at each physiological stage are reported in Table 2SM.

Cortisol concentrations were influenced by baseline values (*p* < 0.05) and showed marked temporal fluctuations, peaking at parturition (*p* < 0.001; Figure 2a; Table 1SM). Although cortisol concentrations declined by day 18 of lactation and at weaning, they remained significantly higher than those observed at AI. A significant group × time interaction was detected (*p* = 0.001). However, post hoc pairwise comparisons identified only two borderline differences after adjustment for multiple testing: LPP animals tended to exhibit higher cortisol concentrations than CNT animals at parturition (*p* = 0.051), whereas CNT animals tended to show higher concentrations than LPP animals at weaning (*p* = 0.055; Table 2SM). These results indicate suggestive trends rather than confirmed treatment effects.

Leptin concentrations also varied significantly over time, decreasing at parturition compared to AI and reaching a peak on day 18 of lactation (*p* < 0.05; Figure 2b; Table 1SM). The group × time interaction was significant (*p* = 0.036). At parturition, leptin concentrations were lower in LPP than in CNT does (*p* < 0.01; Figure 2b; Table 2SM).

Insulin concentrations were significantly affected by group, time, and their interaction (all *p* < 0.05; Table 1SM). Insulin levels decreased after parturition, with the lowest marginal means observed at weaning (*p* < 0.01). Overall, the L group showed higher insulin concentrations than the CNT group (*p* < 0.01). At day 18 of lactation, insulin concentrations were higher in both supplemented groups than in CNT does (all *p* < 0.05). At weaning, insulin concentrations remained higher in L than in CNT (*p* = 0.002) and in L than in LPP (*p* < 0.001; Figure 2c; Table 2SM).

The marginal means of T_3_ progressively increased from artificial insemination to weaning (*p* < 0.001; Figure 2d; Table 1SM). Dietary treatment also had a significant effect (*p* < 0.001), with the highest values observed in the LPP group, followed by the L and CNT groups. A significant treatment × stage interaction was detected (*p* < 0.01). Compared with CNT does, LPP does showed higher T_3_ concentrations at AI (*p* < 0.01), parturition (*p* < 0.001), day 18 of lactation (*p* = 0.001), and weaning (*p* < 0.05). T_3_ concentrations were also higher in the LPP group than in the L group at parturition (*p* = 0.001), whereas differences between these groups at the other physiological stages did not reach statistical significance (Table 2SM).

T_4_ concentrations were influenced only by the main effects of group and time (*p* < 0.01; Table 1SM). The LPP group showed higher marginal means than the L group. Over time, T_4_ levels reached a nadir at parturition, followed by a progressive increase throughout lactation (*p* < 0.05). Pairwise comparisons revealed higher T4 concentrations in the LPP group than in the L group at AI and at parturition (all *p* < 0.05; Figure 2e; Table 2SM).

The T_3_/T_4_ ratio was significantly affected by dietary treatment, physiological stage, and their interaction (all *p* < 0.01), with the lowest marginal means observed in the CNT group and at artificial insemination (*p* < 0.05). Pairwise comparisons revealed a higher T3/T4 ratio in the LPP group than in the CNT group at AI (*p* < 0.05), parturition (*p* < 0.01), and day 18 of lactation (*p* < 0.01). At AI, the L group also exhibited a higher T3/T4 ratio than the CNT group (*p* < 0.001; Figure 2f; Table 2SM).

**Figure 2.**
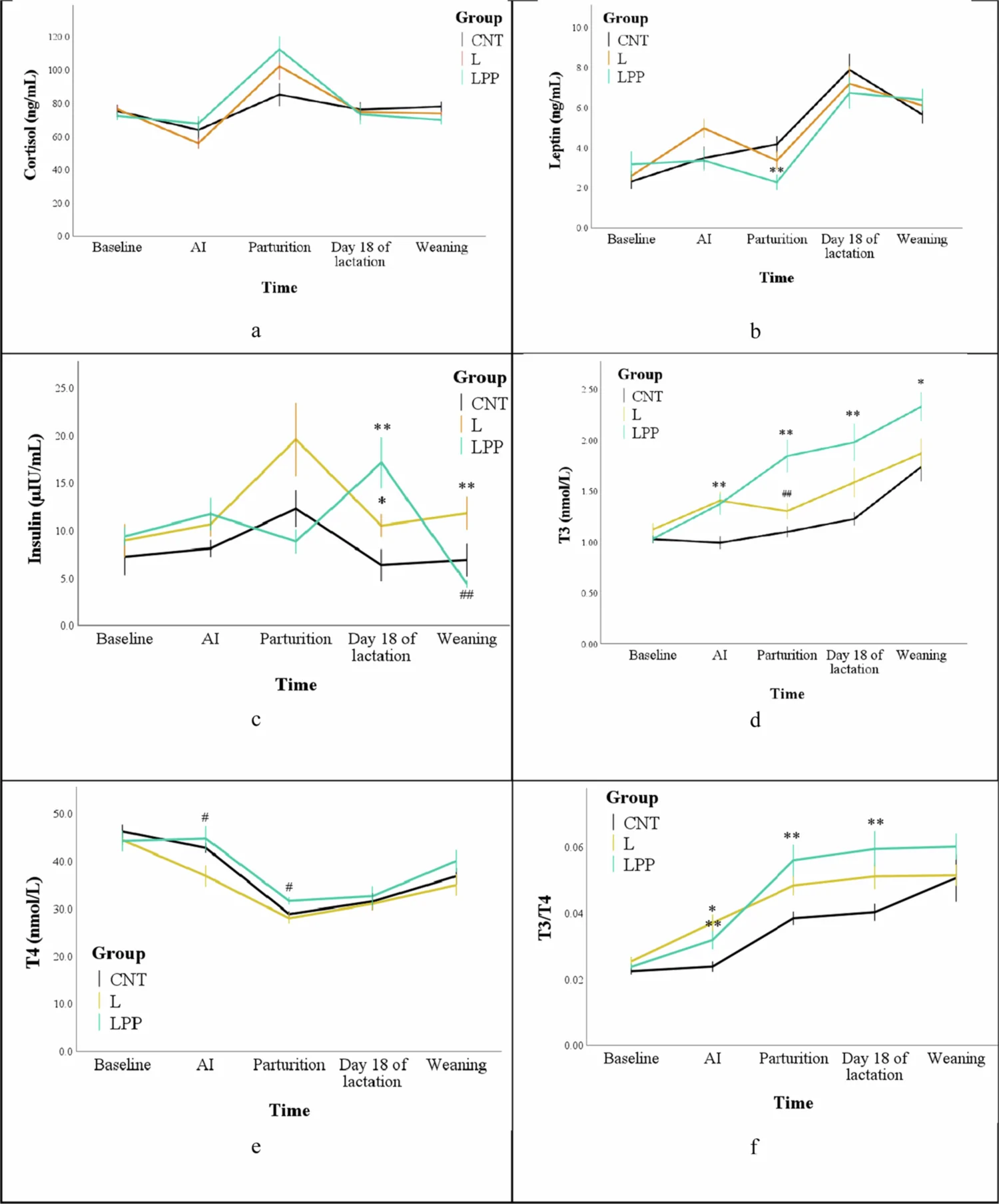
Effects of the experimental diets on plasma cortisol, leptin, insulin, thyroid hormones (T₃ and T₄), and the T₃/T₄ ratio in rabbit does throughout the first reproductive cycle. CNT: control diet; L: diet supplemented with 5% extruded linseed; LPP: diet supplemented with 5% extruded linseed and 0.2% *Padina pavonica* extract. Blood samples were collected at baseline (before dietary adaptation), artificial insemination (AI), parturition, mid-lactation (18 days postpartum), and weaning (35 days postpartum). Values are presented as means ± standard errors. Asterisks indicate significant differences compared to the control group (**p < 0.01, *p < 0.05), whereas hash symbols indicate differences between L and LPP groups (^##^ *p* < 0.01, ^#^ *p* < 0.05).

NEFA concentrations were significantly affected by physiological stage (*p* < 0.001), but not by dietary treatment or the treatment × stage interaction (Table 1SM). The marginal means of NEFA concentrations increased markedly from AI to parturition and remained elevated throughout lactation (*p* < 0.001). At AI, NEFA concentrations were lower in the LPP group than in the CNT group (*p* < 0.05), while no further differences among groups were detected (Figure 3a; Table 2SM).

Glucose concentrations were affected exclusively by sampling time (*p* < 0.001; Table 1SM). Concentrations decreased significantly from AI to parturition in all groups, remained stable from parturition through day 18 of lactation, and declined again at weaning (*p* < 0.01; Figure 3b). No significant differences among dietary groups were detected (Table 2SM).

**Figure 3.**
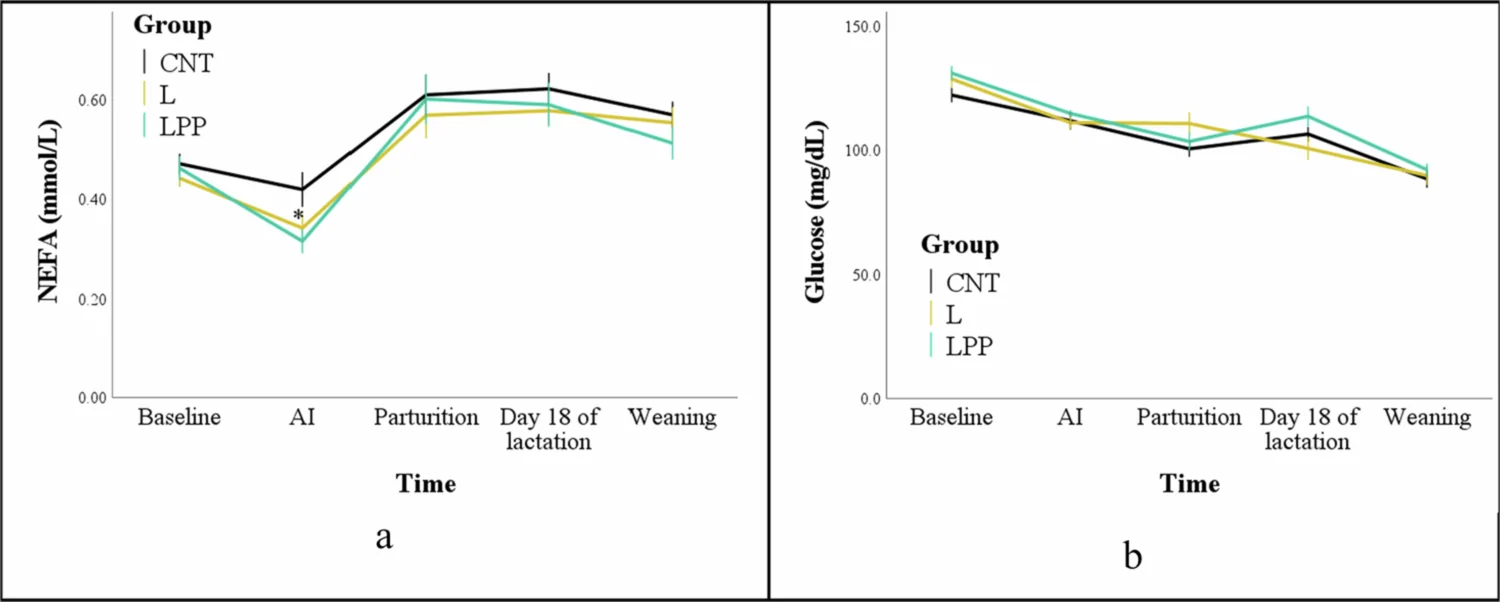
Effects of the experimental diets on plasma non-esterified fatty acids (NEFA) and glucose concentrations in rabbit does throughout the first reproductive cycle. CNT: control diet; L: diet supplemented with 5% extruded linseed; LPP: diet supplemented with 5% extruded linseed and 0.2% *Padina pavonica* extract. Blood samples were collected at baseline (before dietary adaptation), artificial insemination (AI), parturition, mid-lactation (18 days postpartum), and weaning (35 days postpartum).Values are presented as means ± standard errors. Asterisks indicate significant differences compared to the control group (**p < 0.01, *p < 0.05), while hash symbols indicate differences between L and LPP groups (^##^ *p* < 0.01, ^#^ *p* < 0.05).

### 3.2. Associations between hormones and metabolites across physiological stages

Correlations between hormones and metabolites across the different physiological stages are reported in Table 4. Regardless of the nutritional group, at the various time points, several significant associations were observed. Cortisol showed a moderate negative correlation with T₃ at the time of AI (*p* < 0.05), whereas this correlation became positive both at parturition and on day 18 of lactation (*p* < 0.01). Leptin was moderately negatively correlated with T₃ (*p* < 0.05) and with blood glucose (*p* < 0.01) at parturition. Insulin exhibited a strong positive correlation with blood glucose at baseline (*p* < 0.01), and negative correlations with NEFA at AI (*p* < 0.01) and with T₄ at parturition (*p* < 0.05). In addition to its previously described associations with cortisol and leptin, T₃ showed a moderate negative correlation with NEFA and moderate positive correlations with both T₄ and glucose on day 18 of lactation (*p* < 0.05).

**Table 4 .** Pearson’s correlation coefficient between hormones and metabolites across the different physiological stages.

Physiological stage			Leptin (ng/mL)	Insulin (μIU/mL)	T_3_(nmol/L)	T_4_(nmol/L)	NEFA (mmol/L)	Glucose (mg/dL)
Baseline	Cortisol (ng/mL)		.010	−.058	.167	.113	−.064	−.227
	Leptin (ng/mL)			−.114	−.154	−.272	.052	.004
	Insulin (μIU/mL)				.130	−.116	−.142	**.535** ^**^
	T3 (nmol/L)					.246	−.241	−.061
	T4 (nmol/L)						−.201	−.294
	NEFA (mmol/L)							.025
AI	Cortisol (ng/mL)		−.041	−.167	−**.310** ^*^	.092	.167	−.084
	Leptin (ng/mL)			.152	.209	−.031	−.240	.156
	Insulin (μIU/mL)				.235	−.085	−**.436** ^**^	−.038
	T3 (nmol/L)					−.085	−.140	−.106
	T4 (nmol/L)						−.086	.098
	NEFA (mmol/L)							−.022
Parturition	Cortisol (ng/mL)		−.274	−.088	**.394** ^**^	−.066	.320^*^	.129
	Leptin (ng/mL)			.095	−**.336** ^*^	−.259	−.071	−**.382** ^**^
	Insulin (μIU/mL)				−.196	−**.339** ^*^	−.042	.126
	T3 (nmol/L)					.260	.037	.168
	T4 (nmol/L)						.024	.185
	NEFA (mmol/L)							.014
Day 18 of lactation	Cortisol (ng/mL)		.195	.138	**.457** ^**^	.126	−.263	.171
	Leptin (ng/mL)			−.190	−.031	.074	−.003	−.067
	Insulin (μIU/mL)				.285	.004	−.005	.111
	T3 (nmol/L)					**.375** ^*^	−**.372** ^*^	**.331** ^*^
	T4 (nmol/L)						−.010	.224
	NEFA (mmol/L)							−.295
Weaning	Cortisol (ng/mL)		−.164	.208	−.254	−.185	.061	**.346** ^*^
	Leptin (ng/mL)			−.031	.027	.113	−.157	.068
	Insulin (μIU/mL)				−.236	−.244	.114	.134
	T3 (nmol/L)					.135	−.035	−.122
	T4 (nmol/L)						−.117	**.371** ^*^
	NEFA (mmol/L)							−.284

Statistically significant coefficients are in bold (**p < 0.01, *p < 0.05).

### 3.3. Hormonal and metabolic profiles characterizing nutritional treatments and physiological stages

Figure 4 shows discriminant scores and centroids of the two discriminant functions (DF1 and DF2) according to nutritional treatment (DA1). Details of the structure matrix and centroids at the different time points are presented in Tables 3SM and 4SM. Cortisol and glucose were not included in this DA because they were not significant in the equality of group means test, indicating that they were not affected by the nutritional treatment. The DA1 extracted a first DF that explained approximately 70% of the variance and was defined by a strong positive coefficient for T_3_ (0.661), a moderate positive coefficient for insulin (0.455), and a pronounced negative coefficient for NEFA (−0.601). DF2 was instead only characterized by a positive coefficient for leptin (0.926). The CNT group showed a strongly negative DF1 centroid and a moderately negative DF2 centroid, suggesting an association with low T_3_ and elevated NEFA. By contrast, both *n*-3 PUFA-supplemented groups (L and LPP) had positive DF1 centroids, indicating higher T_3_ and lower NEFA. These two groups, however, were distinguished by DF2: the L group exhibited a positive DF2 centroid, whereas the LPP group’s DF2 centroid was negative, indicating higher leptin concentrations in the L group compared to the LPP group.

**Figure 4.**
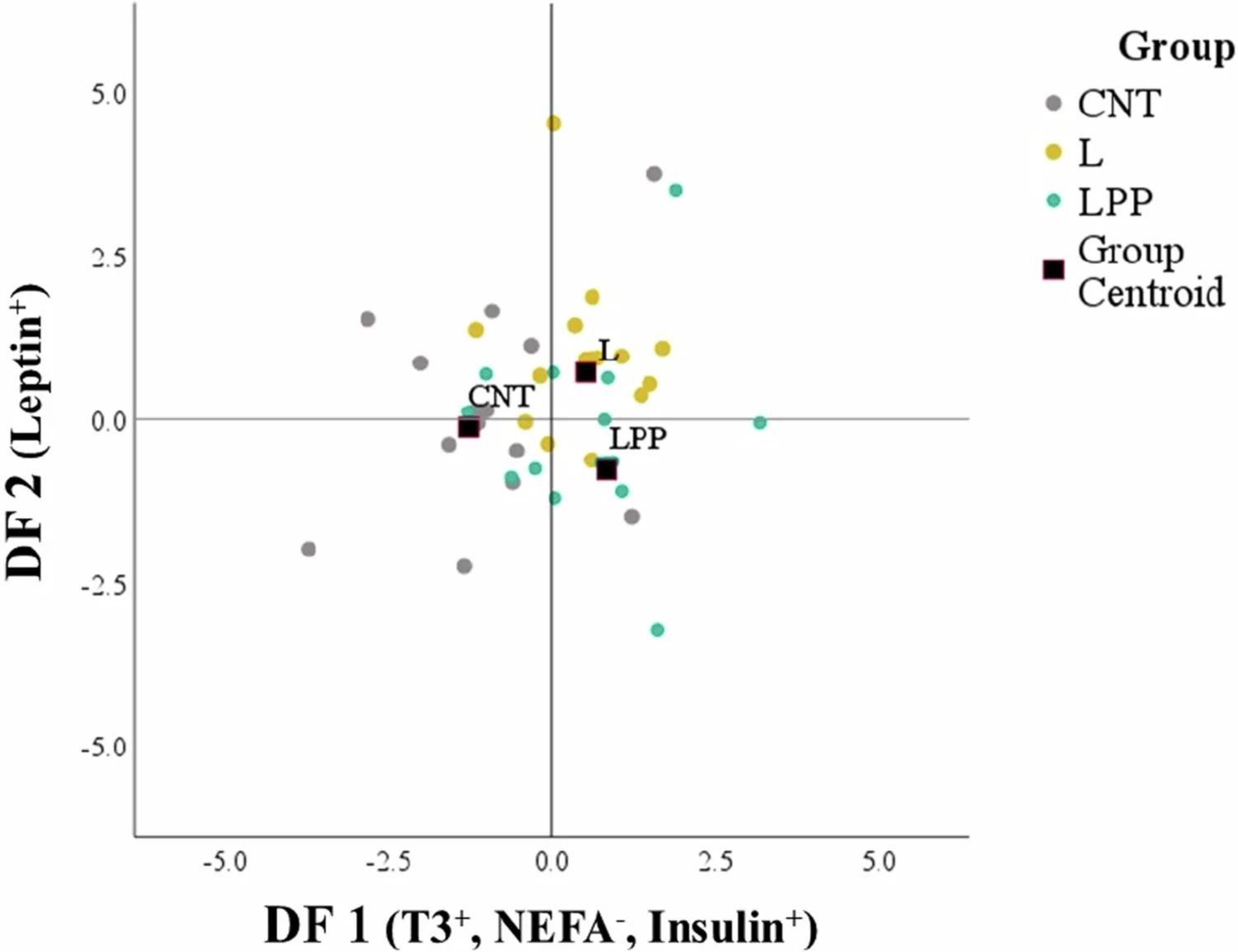
Discriminant scores for Discriminant Function (DF) 1 and 2 with centroids according to nutritional group at Artificial insemination. CNT, control diet; L, diet supplemented with 5% extruded linseed; LPP, diet supplemented with 5% extruded linseed and 0.2% *Padina pavonica* extract. The sign of the coefficient for variables exhibiting the largest absolute correlation with their respective DF is indicated in parentheses: DF1 loaded positively mainly for T_3_ and negatively for NEFA; DF2 loaded positively for leptin.

In Figure 5, the discriminant scores and their centroids for DF1–DF3 are plotted across the various physiological stages (DA2). The complete structure‐matrix loadings and centroid values for each time point are reported in Tables 5SM and 6SM. Briefly, DF1 had high positive coefficients for leptin (0.648) and T_3_ (0.407), DF2 for cortisol (0.806) and NEFA (0.510), and DF3 for glucose (0.655; Table 5SM). At AI, both DF1 and DF2 were negative, indicating low leptin, T_3_, cortisol, and NEFA (Figure 5a). Parturition was characterized predominantly by positive DF2 values, consistent with relatively higher cortisol and NEFA levels (Figure 5a). Both day 18 of lactation and weaning exhibited clearly positive DF1 centroids and DF2 centroids just below zero, indicating higher leptin and T_3_ concentrations together with lower cortisol and NEFA levels compared to parturition (Figure 5a). However, the two lactation time points were distinguished by distinct centroids for DF3: it was positive at 18 days, indicating high circulating glucose, and negative at weaning (Figure 5b).

**Figure 5.**
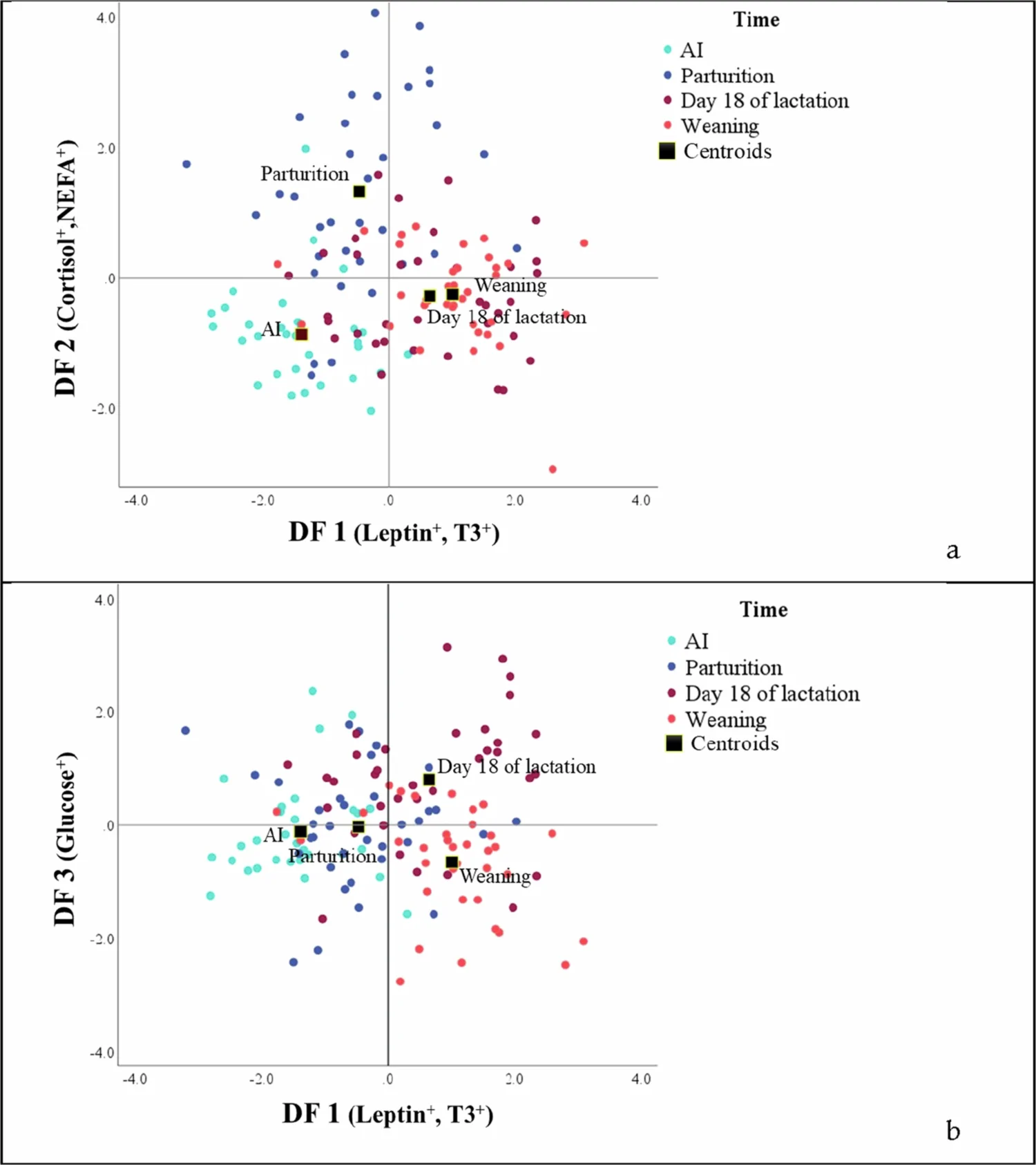
Discriminant analysis of endocrine and metabolic profiles across physiological stages. Discriminant scores for Discriminant Function (DF) 1 and 2 (panel a) and for Discriminant Function 1 and 3 (panel b), with centroids according to physiological stages. The sign of the coefficient for variables exhibiting the largest absolute correlation with their respective DF is indicated in parentheses: DF1 loaded positively on leptin and T_3_; DF2 on cortisol and NEFA; DF3 on glucose.

## 4. Discussion

The present study investigated the metabolic and endocrine adaptations occurring in nulliparous rabbit does receiving different dietary treatments throughout their first reproductive cycle. As expected, the endocrine and metabolic profiles varied according to reproductive stage, reflecting the dynamic physiological demands associated with mating, gestation, parturition, and lactation. In addition, dietary supplementation with *n*-3 PUFA, provided with extruded linseed (L) either alone or combined with *Padina pavonica* extract (LPP), was associated with changes in the selected endocrine and metabolic indicators, particularly thyroid hormone concentrations, insulin and leptin dynamics, and lipid mobilization. These findings suggest that incorporating *n*-3 PUFA and algal bioactives might play a supportive modulatory role in the doe's metabolic adaptation during the intense physiological demands of reproduction.

Cortisol concentrations were primarily influenced by the reproductive stage, reaching their highest values at parturition. This pattern is consistent with the well-established activation of the hypothalamic–pituitary–adrenal axis during late gestation, which plays a central role in the endocrine cascade leading to parturition and contributes to fetal lung maturation (Menchetti et al. 2020; Benedek et al. 2021). Following parturition, cortisol concentrations declined, although they remained moderately elevated throughout lactation. This sustained cortisol release may reflect the continued metabolic demands associated with milk production, consistent with the recognized role of glucocorticoids in mammary development, lactogenesis, and the maintenance of lactation (Bigler et al. 2023). Although a significant group × time interaction was detected, differences between dietary groups were not confirmed by the adjusted post hoc comparisons. Therefore, the interaction suggests that cortisol concentrations may have followed different temporal patterns among groups, but does not provide sufficient evidence to support a specific dietary effect on circulating cortisol concentrations. This interpretation is consistent with our previous observations in growing rabbits, in which dietary supplementation with extruded linseed, either alone or in combination with *Padina pavonica*, did not significantly modify circulating cortisol concentrations despite influencing other endocrine and metabolic variables (Quattrone et al. 2025). Although *n*-3 PUFA have been reported to modulate glucocorticoid responses under specific experimental or stress-related conditions in other species, these effects appear to depend on the physiological context and the nature of the stressor rather than representing a generalized effect on circulating cortisol concentrations (Madison et al. 2021; Nemeth et al. 2021). Therefore, the absence of significant dietary effects observed in the present study suggests that the physiological endocrine changes associated with gestation, parturition, and lactation were the predominant determinants of circulating cortisol concentrations under the experimental and physiological conditions evaluated.

Leptin concentrations also varied according to reproductive stage, decreasing at parturition and increasing during mid-lactation. This temporal pattern is consistent with previous observations in rabbit does and reflects the profound metabolic adjustments accompanying the transition from gestation to lactation, during which maternal energy reserves are mobilized to support milk production (Manal et al. 2010; Peiró et al. 2023). Lower circulating leptin concentrations around parturition are also considered part of the physiological adaptation that facilitates the increase in voluntary feed intake required to meet the energetic demands of lactation (Woodside et al. 2000; Menchetti et al. 2020). The subsequent increase observed during mid-lactation may additionally reflect the increased expression of leptin within the mammary gland during lactation. Indeed, leptin is synthesized by rabbit mammary epithelial cells, and its expression increases significantly between days 3 and 16 of lactation, suggesting an autocrine or paracrine role in mammary development and function as well as a potential contribution to neonatal metabolic regulation through milk (Bonnet et al. 2002; Koch et al. 2013; Kirat et al. 2015). Beyond these physiological changes, does receiving the LPP diet exhibited significantly lower circulating leptin concentrations at parturition than control animals. Brown macroalgae are increasingly recognized for their anti-obesity and metabolic regulatory properties, which have been demonstrated in *in vitro* studies, rodent models, and, to a lesser extent, in human clinical investigations (Magwaza and Islam 2023; Zarrin and Taherizadeh 2025; Pang et al. 2026). Their bioactive constituents, including phlorotannins, sulfated polysaccharides, carotenoids, and polyphenols, have been associated with reduced adipose tissue accumulation, modulation of adipokine secretion, attenuation of adipose inflammation, and improvements in insulin sensitivity through pathways involving AMP-activated protein kinase (AMPK), peroxisome proliferator-activated receptors (PPARs), and inflammatory signaling cascades (Khalfoun et al. 1997; Karbowska and Kochan 2005; Gray et al. 2013). Although these findings provide biologically plausible mechanisms that may explain the lower leptin concentrations observed in the LPP group, comparable evidence is currently lacking in rabbits and, to our knowledge, no studies have specifically investigated the effects of *Padina pavonica* supplementation on leptin regulation during reproduction.

Insulin concentrations varied according to both reproductive stage and dietary treatment. In control animals, insulin concentrations declined after parturition and reached their lowest values at weaning, consistent with the physiological transition from the anabolic state of pregnancy, characterized by increased insulin secretion and relative insulin resistance to support fetal growth (Menchetti et al. 2020; Brecchia et al. 2021), to the predominantly catabolic state of lactation, during which nutrients are preferentially partitioned toward milk synthesis (Nommsen-Rivers 2016). During this period, blood glucose is preferentially diverted to the mammary gland for lactose synthesis, resulting in lower systemic insulin secretion while maintaining selective peripheral insulin resistance to ensure nutrient availability for milk production (Ladyman and Brooks 2021). Interestingly, dietary *n*-3 PUFA supplementation was associated with modifications of this physiological pattern. Does receiving the L diet maintained relatively higher insulin levels from parturition to late lactation, alongside normal glycemia and reduced NEFA, suggesting improved metabolic control and reduced lipid mobilization (Rhee and Brunt 2011; Mohammadi-Sartang et al. 2018; Seliem et al. 2022). The concurrent euglycemia and effective suppression of NEFA reflect the preserved antilipolytic action of insulin, thereby confirming maintained peripheral insulin sensitivity. Conversely, the LPP does showed a transient insulin peak at mid-lactation, coinciding with the period of peak milk production in rabbits (Ludwiczak et al. 2023). This temporary rise may reflect metabolic adjustments required to support the high glucose turnover associated with maximal lactose synthesis (Draganescu et al. 2021; Nowak and Jeziorek 2023). By the end of lactation, insulin concentrations declined in all groups but remained lowest in the LPP group, supporting the hypothesis that algal supplementation may improve long-term insulin efficiency and sensitivity due to its antidiabetic properties (Lalia and Lanza 2016; Germoush et al. 2019; Akl et al. 2025). This effect may be related to the presence of long-chain *n*-3 PUFA together with bioactive compounds present in *Padina pavonica*, which have been reported to enhance glucose uptake, improve insulin signaling, and reduce oxidative and inflammatory stress (Pan et al. 2007; Mohammadi-Sartang et al. 2018).

The metabolite profiles closely paralleled the endocrine changes observed throughout the reproductive cycle. Regardless of dietary treatment, circulating NEFA concentrations increased markedly from artificial insemination to parturition and remained elevated throughout lactation, reflecting the progressive mobilization of maternal lipid reserves to meet the increasing energetic demands of fetal growth and milk production (Fortun-Lamothe 2006; Menchetti et al. 2020). This pattern is consistent with previous studies in rabbit does, in which late gestation and early lactation are characterized by a negative energy balance and increased utilization of body fat reserves, particularly in primiparous animals (Rodríguez et al. 2017; García et al. 2019; Menchetti et al. 2020). Interestingly, does receiving the LPP diet exhibited lower circulating NEFA concentrations at artificial insemination than control animals, suggesting reduced lipid mobilization at this early stage of the reproductive cycle. Similar effects have been reported for brown macroalgae in experimental models, where their bioactive constituents have been shown to reduce circulating free fatty acids through modulation of adipose tissue lipolysis, stimulation of fatty acid oxidation, and regulation of lipid metabolism (Farsi et al. 2014; Jump et al. 2018; Yuan et al. 2020; Pandey et al. 2025).

Concurrently, blood glucose concentrations remained within the physiological range throughout the study and followed the expected temporal pattern, reaching their lowest values at parturition before increasing during lactation. Similar fluctuations have been reported in rabbit does and are generally attributed to the increased maternal and fetal utilization of glucose during late gestation, followed by the maintenance of glucose availability to support lactose synthesis during lactation (Hauguel et al. 1987; Menchetti et al. 2015, 2020). The absence of significant differences among dietary groups was expected, and it possibly reflects an effective glycemic regulation despite intense physiological demands.

Thyroid hormones further highlighted these metabolic adaptations during the reproductive cycle. Maternal thyroid activity supports metabolic adaptation during pregnancy and exerts a galactopoietic effect during lactation by stimulating mammary metabolism and nutrient partitioning toward milk synthesis (Capuco et al. 2008). These functions are particularly important in primiparous rabbit does, which must simultaneously sustain their own body growth while supporting gestation and milk production (Menchetti et al. 2015). In the present study, circulating T_3_ concentrations progressively increased from artificial insemination to weaning, whereas T_4_ declined at parturition before increasing during lactation, potentially suggesting enhanced peripheral conversion of T_4_ into the biologically active T_3_ as metabolic demands increased. This response was amplified in supplemented groups, particularly LPP, which showed the highest T_3_ concentrations and T_3_/T_4_ ratio. The inclusion of *Padina pavonica*, which contains iodine and other bioactive compounds such as polysaccharides and polyphenols, which are known to support thyroid function (Ahmed et al. 2024; Quattrone et al. 2025; Cadar et al. 2025), may have potentially contributed to these differences. However, this interpretation remains hypothetical because iodine status and deiodinase activity were not directly assessed in the present study. The effects of dietary *n*-3 PUFA on circulating thyroid hormones remain somewhat controversial in the existing literature, with some studies reporting neutral effects on basal activity (Benvenga et al. 2022) and others suggesting improved receptor sensitivity (Souza et al. 2010). Thus, the higher T₃ concentrations and T₃/T₄ ratio observed in the supplemented groups, particularly LPP, may reflect a potential interaction between *n*-3 PUFA and the algal component of the diet. One possible explanation is that this combination might facilitate enhanced peripheral deiodinase activity (the conversion of T₄ to T₃), thereby upregulating the metabolic machinery required to sustain the intense energetic demands of lactation while sparing body reserves (Russo et al. 2021). This proposed mechanism could effectively upregulate the metabolic machinery required to sustain the intense energy demands of lactation, ultimately helping to spare maternal body reserves. However, this mechanism remains speculative and cannot be confirmed from the present data. Further studies assessing iodine status, thyroid function, and deiodinase activity are therefore warranted to determine the mechanisms underlying the observed thyroid hormone responses.

The complex interactions between hormonal and metabolic indicators were further explored through correlation analysis. At baseline, the positive glucose-insulin relationship confirmed normal glycemic regulation in non-pregnant does (Rommers et al. 2004). At this stage, the rabbits were not yet exposed to the metabolic demands associated with gestation or lactation, and insulin secretion likely responded directly to circulating glucose levels to maintain metabolic homeostasis (Röder et al. 2016). At artificial insemination, insulin was negatively correlated with NEFA, a finding consistent with the well-established antilipolytic action of insulin (Chakrabarti et al. 2013). In addition, cortisol was inversely associated with T₃, suggesting an interaction between stress and thyroid axes, as glucocorticoids can inhibit the peripheral conversion of T₄ to T₃ and suppress overall thyroid activity (Haugen 2009; Nadolnik 2012). However, at parturition and mid-lactation, cortisol and T_3_ became positively correlated, highlighting the simultaneous activation of stress and metabolic pathways required for kindling and milk synthesis (Motil et al. 1994). Furthermore, negative correlations between leptin, T₃, and glucose at parturition further indicate the metabolic reorganization toward lactation (Menchetti et al. 2020). Particularly, negative correlations between T_3_ and NEFA during peak lactation suggest that thyroid-driven oxidative metabolism successfully reduced the need for excessive lipid mobilization (Chakrabarti et al. 2013) cycle. Concurrently, the positive correlations between T₃, T₄, and glucose likely reflect the elevated metabolic turnover required to sustain lactation (Rommers et al. 2004).

Discriminant analyses provided an integrated overview of how these distinct endocrine profiles were influenced by both dietary treatment and physiological stage. Importantly, because discriminant functions identify patterns of multivariate separation among groups, these results should be considered exploratory rather than evidence of direct causal relationships among variables. Regarding dietary treatment, the supplemented groups were primarily characterized by higher circulating T_3_ concentrations and lower NEFA values than the control group, suggesting a higher basal metabolism coupled with reduced lipid mobilization. These findings align with the established metabolic effects of *n*-3 PUFA, which enhance oxidative metabolism and improve insulin signaling while reducing circulating fatty acids (Baynes et al. 2018). Furthermore, the DA separated the two supplemented groups primarily based on leptin, with the linseed-only (L) group exhibiting higher concentrations than the LPP group. While elevated leptin is often linked to increased adiposity, our previous evaluation of these exact diets revealed no significant differences in body weight among groups (Quattrone et al. 2024). Therefore, we hypothesize that this difference is more likely driven by endocrine cross-talk. The higher leptin in the L group may be a consequence of their sustained insulin concentrations, as insulin is a known stimulator of adipocyte leptin synthesis and secretion (Dagogo-Jack 2024). Conversely, the lower leptin levels in the LPP group suggest that the additional bioactive compounds in the brown algae might enhance peripheral leptin sensitivity (Can and Sanlier 2026). This improved sensitivity would theoretically allow the LPP does to optimize energy utilization and maintain metabolic homeostasis without the need for high circulating leptin levels. When evaluated across the reproductive timeline, the models confirmed highly stage-dependent profiles. Artificial insemination was characterized by low leptin, T_3_, cortisol, and NEFA, reflecting a basal, non-pregnant state. Parturition was distinctly dominated by stress-related endocrine activity and acute lipid mobilization (elevated cortisol and NEFA) to initiate lactation (Minuti et al. 2015). Conversely, mid-lactation and weaning were defined by metabolic stabilization, indicated by higher leptin and T_3_ alongside lower cortisol and NEFA, as the animals likely shifted toward more efficient nutrient utilization. The primary distinction between these two late stages was circulating glucose, which peaked at mid-lactation to support massive lactose synthesis before progressively declining by weaning as lactational demands decrease (Capuco et al. 2008).

Finally, although litter size represents a potential source of biological variability, previous studies have shown that its influence on maternal metabolism is greatest during late gestation and becomes less pronounced after parturition (Minuti et al. 2015, 2020). Moreover, since mean litter size was comparable among the experimental groups, we believe that the potential influence of this biological variable on the endocrine and metabolic responses observed during both late pregnancy and lactation was likely minimized. Another limitation concerns the statistical power to detect small treatment effects. Although the a priori power analysis indicated that the sample size was adequate to detect medium-sized effects, the evaluation of multiple biomarkers across several physiological stages and adjustment for multiple comparisons may have reduced the ability to detect smaller differences. Therefore, the possibility of Type II errors cannot be excluded, and non-significant findings should not necessarily be interpreted as evidence of the absence of dietary effects. Larger studies are warranted to confirm subtle treatment differences and establish their biological relevance.

From a practical perspective, the inclusion of functional ingredients such as extruded linseed and *Padina pavonica* extract may represent a promising nutritional strategy for breeding rabbit does, particularly during metabolically demanding reproductive stages. The endocrine and metabolic adaptations observed in the supplemented groups, including higher thyroid activity, improved insulin dynamics, and reduced lipid mobilization, suggest that these dietary interventions may support a more efficient physiological adaptation to the energetic demands of reproduction. Supporting these adaptive mechanisms through targeted nutritional management is consistent with current approaches aimed at improving animal welfare, reproductive performance, and the long-term health of breeding females. Nevertheless, larger-scale field studies under commercial production conditions are required to determine whether these endocrine and metabolic responses translate into sustained improvements in doe longevity, welfare, reproductive success, and production efficiency.

## 5. Conclusions

In conclusion, dietary supplementation with *n*-3 PUFA, particularly when extruded linseed was combined with *Padina pavonica* extract, was associated with changes in thyroid hormone concentrations, insulin dynamics, leptin concentrations, and lipid mobilization in nulliparous rabbit does during their first reproductive cycle. The higher circulating T_3_ concentrations and lower NEFA levels observed in the supplemented groups suggest that these dietary strategies may influence endocrine and metabolic adaptations during physiologically demanding reproductive stages. Although the present findings suggest that functional dietary supplementation may influence endocrine and metabolic profiles in breeding rabbit does, the mechanisms underlying these responses, including the proposed effects on thyroid function, remain hypothetical and require further investigation. Future studies are therefore needed to elucidate the mechanisms involved and determine their physiological and practical relevance.

Future studies should also evaluate whether these responses are maintained under the more demanding reproductive conditions typical of commercial rabbit production, particularly in primiparous and multiparous does experiencing overlapping gestation and lactation. In addition, integrating endocrine and metabolic profiling with molecular biomarkers and long-term productive outcomes, including milk yield, offspring growth, reproductive longevity, and lifetime doe performance, will be essential to clarify the mechanisms involved and determine the practical relevance of these nutritional strategies. Finally, although the sample size provided adequate statistical power to detect medium-sized effects, subtle treatment differences may have gone undetected, particularly after adjustment for multiple comparisons. Therefore, larger longitudinal studies are warranted to validate and extend the present findings.

## Supplementary Material

SM_R3_DEF.docx

## Data Availability

The authors confirm that the data supporting the findings of this study are available within the article and its supplementary materials.
