# Supplementary material for: Hormonal and metabolic adaptations in Omega-3-supplemented rabbit does during their first reproductive cycle
Source: Vet Q. 2026 Oct 29;46(1):2749322. doi: 10.1080/01652176.2026.2749322 (PMC13721818; doi:10.1080/01652176.2026.2749322)
Supplement: Supplementary file 1 [file TVEQ_A_2749322_SM2680.docx]

**Supplementary material**

**Table 1SM.** Effects of dietary treatment, physiological stage, and their interaction on hormonal and metabolic variables in rabbit does.

| **Variable** | **Basal levels** | **Dietary treatment** | **Physiological stage** | **Treatment × stage** |
| --- | --- | --- | --- | --- |
| Cortisol | 0.024 | 0.245 | <0.001 | 0.001 |
| Leptin | 0.911 | 0.316 | 0.036 | 0.036 |
| Insulin* | 0.049 | 0.003 | 0.004 | 0.003 |
| T3 | 0.523 | <0.001 | <0.001 | 0.003 |
| T4 | 0.877 | 0.002 | <0.001 | 0.532 |
| T3/T4 ratio | 0.905 | <0.001 | <0.001 | 0.003 |
| NEFA | 0.191 | 0.208 | <0.001 | 0.591 |
| Glucose | 0.571 | 0.293 | <0.001 | 0.117 |

Values are *p*-values obtained from the generalized linear mixed models. NEFA, non-esterified fatty acids.

* After ln transformation

**Table 2SM. Pairwise comparisons among dietary treatments for hormonal and metabolic variables at each physiological stage. Results are reported as estimated mean differences (95% confidence intervals [CI]) with corresponding adjusted p-values. NS indicates pairwise comparisons that did not reach statistical significance (p > 0.05). CNT, control diet; L, diet supplemented with 5% extruded linseed; LPP, diet supplemented with 5% extruded linseed and 0.2% *Padina pavonica* extract; AI, artificial insemination.**

| **Variable** | **Stage** | **L vs CNT** | **LPP vs CNT** | **LPP vs L** |
| --- | --- | --- | --- | --- |
| **Cortisol** | AI | NS | NS | NS |
|  | Parturition | NS | 26.749 (−0.098 to 53.596); *p* = 0.051 | NS |
|  | Day 18 lactation | NS | NS | NS |
|  | Weaning | NS | −8.490 (−17.122 to 0.143); *p* = 0.055 | NS |
| **Leptin** | AI | NS | NS | NS |
|  | Parturition | NS | −1.902 (−3.412 to −0.391); *p* = 0.008 | NS |
|  | Day 18 lactation | NS | NS | NS |
|  | Weaning | NS | NS | NS |
| **Insulin†** | AI | NS | NS | NS |
|  | Parturition | NS | NS | NS |
|  | Day 18 lactation | 0.699 (0.122 to 1.276); *p* = 0.014 | 1.035 (0.416 to 1.654); *p* < 0.001 | NS |
|  | Weaning | 0.633 (0.207 to 1.058); *p* = 0.002 | NS | −0.940 (−1.388 to −0.493); *p* < 0.001 |
| **T3 (nmol/L)** | AI | NS | 0.400 (0.122 to 0.679); *p* = 0.003 | NS |
|  | Parturition | NS | 0.739 (0.359 to 1.118); *p* < 0.001 | 0.573 (0.215 to 0.931); *p* = 0.001 |
|  | Day 18 lactation | NS | 0.783 (0.291 to 1.275); *p* = 0.001 | NS |
|  | Weaning | NS | 0.604 (0.089 to 1.119); *p* = 0.016 | NS |
| **T4 (nmol/L)** | AI | NS | NS | 7.881 (0.788 to 14.975); *p* = 0.024 |
|  | Parturition | NS | NS | 3.621 (0.566 to 6.676); *p* = 0.014 |
|  | Day 18 lactation | NS | NS | NS |
|  | Weaning | NS | NS | NS |
| **T3/T4 ratio** | AI | 0.014 (0.006 to 0.022); *p* < 0.001 | 0.009 (0.001 to 0.016); *p* = 0.021 | NS |
|  | Parturition | NS | 0.017 (0.005 to 0.029); *p* = 0.002 | NS |
|  | Day 18 lactation | NS | 0.019 (0.005 to 0.034); *p* = 0.005 | NS |
|  | Weaning | NS | NS | NS |
| **NEFA (mmol/L)** | AI | NS | −0.114 (−0.216 to −0.012); *p* = 0.023 | NS |
|  | Parturition | NS | NS | NS |
|  | Day 18 lactation | NS | NS | NS |
|  | Weaning | NS | NS | NS |
| **Glucose (mg/dL)** | AI | NS | NS | NS |
|  | Parturition | NS | NS | NS |
|  | Day 18 lactation | NS | NS | NS |
|  | Weaning | NS | NS | NS |

Values are estimated mean differences (95% confidence intervals) followed by adjusted p-values. CNT, control diet; L, diet supplemented with 5% extruded linseed; LPP, diet supplemented with 5% extruded linseed and 0.2% *Padina pavonica* extract; AI, artificial insemination. †Insulin differences are expressed on the log-transformed scale.

**Table 3SM**. Structure matrix displaying the discriminant analysis–derived coefficients for hormones and metabolites characterizing the nutritional groups (DA1).

| Variable and variance | Function | |
| --- | --- | --- |
|  | 1 | 2 |
| T3 (nmol/L) | .661^*^ | .159 |
| NEFA (mmol/L) | -.601^*^ | .132 |
| Insulin (µIU/mL) | .455^*^ | -.218 |
| Leptin (ng/mL) | .176 | .926^*^ |
| Variance | 69.8% | 30.2% |

*. Largest absolute correlation between each variable and any discriminant function

**Table 4SM**. Group centroids from discriminant analysis of hormone and metabolite profiles characterizing the nutritional groups (DA1).

| Group | Function | |
| --- | --- | --- |
|  | 1 | 2 |
| CNT | -1.270 | -.119 |
| L | .518 | .721 |
| LPP | .842 | -.776 |

**Table 5SM**. Structure matrix displaying the discriminant analysis–derived coefficients for hormones and metabolites characterizing the physiological stages (DA2).

|  | Function | | |
| --- | --- | --- | --- |
|  | 1 | 2 | 3 |
| Leptin (ng/mL) | .648^*^ | -.352 | .424 |
| T3 (nmol/L) | .407^*^ | -.051 | -.349 |
| Cortisol (ng/mL) | .081 | .806^*^ | .017 |
| NEFA (mmol/L) | .430 | .510^*^ | .275 |
| Glucose (mg/dL) | -.416 | -.034 | .655^*^ |
| Insulin (µIU/mL) | -.096 | .195 | .282^*^ |
| Variance | 48.4% | 34.0% | 17.6% |

*. Largest absolute correlation between each variable and any discriminant function

**Table 6SM**. Group centroids from discriminant analysis of hormone and metabolite profiles characterizing the physiological stages (DA2).

| Time | Function | | |
| --- | --- | --- | --- |
|  | 1 | 2 | 3 |
| AI | -1.608 | -.920 | -.083 |
| Parturition | -.512 | 1.439 | -.039 |
| Day 18 of lactation | .755 | -.279 | .902 |
| Weaning | 1.014 | -.302 | -.780 |
